# Erratum to: Genistein-induced Anticancer Effects on Acute Leukemia Cells Involve the Regulation of Wnt Signaling Pathway Through H4K20me1 Rather Than DNA Demethylation

**DOI:** 10.1007/s11596-021-2478-z

**Published:** 2022-04-18

**Authors:** Hua-rong Zhou, Jian-zhen Shen, Hai-ying Fu, Feng Zhang

**Affiliations:** Fujian Medical Center of Hematology, Fujian Institute of Hematology, Fujian Provincial Key Laboratory of Hematology, Fujian Medical University Union Hospital, Fuzhou, 350001 China

## Erratum

The article “Genistein-induced Anticancer Effects on Acute Leukemia Cells Involve the Regulation of Wnt Signaling Pathway Through H4K20me1 Rather Than DNA Demethylation”, written by Hua-rong ZHOU, Jian-zhen SHEN, Hai-ying FU, Feng ZHANG was originally published electronically on the publisher’s internet portal on October 2021 without open access. With the author(s)’ decision to opt for Open Choice, the copyright of the article is changed to © The Author(s) 2021 and the article is forthwith distributed under a Creative Commons Attribution 4.0 International License (https://creativecommons.org/licenses/by/4.0/), which permits use, sharing, adaptation, distribution and reproduction in any medium or format, as long as you give appropriate credit to the original author(s) and the source, provide a link to the Creative Commons license, and indicate if changes were made.

The original article has been corrected.

